# White rice intake and incidence of type-2 diabetes: analysis of two prospective cohort studies from Iran

**DOI:** 10.1186/s12889-016-3999-4

**Published:** 2017-01-31

**Authors:** Asieh Golozar, Davood Khalili, Arash Etemadi, Hossein Poustchi, Akbar Fazeltabar, Firoozeh Hosseini, Farin Kamangar, Masoud Khoshnia, Farhad Islami, Farzad Hadaegh, Paul Brennan, Paolo Boffetta, Christian C. Abnet, Sanford M. Dawsey, Fereidoun Azizi, Reza Malekzadeh, Goodarz Danaei

**Affiliations:** 1 Digestive Disease Research Institute, Shariati Hospital, Tehran University of Medical Sciences, Tehran, 14117 Iran; 2 Department of Epidemiology, Johns Hopkins Bloomberg School of Public Health, Baltimore, MD 21205 USA; 3 Prevention of Metabolic Disorders Research Center, Research Institute for Endocrine Sciences, Shahid Beheshti University of Medical Sciences, Tehran, 17413 Iran; 4 Division of Cancer Epidemiology and Genetics, National Cancer Institute, Bethesda, MD 20850 USA; 5 Nutrition and Endocrine Research Center/Obesity Research Center,Research Institute for Endocrine Sciences, Shahid Beheshti University of Medical Science, Tehran, 17413 Iran; 6 School of Community Health and Policy, Morgan State University, Baltimore, 21251 MD USA; 7 Golestan Research Center of Gastroenterology and Hepatology, Golestan University of Medical Sciences, Gorgan, Golestan 0619 Iran; 8 Surveillance and Health Services Research, American Cancer Society, Atlanta, 30303 Georgia USA; 9 International Agency for Research on Cancer, Lyon, 69008 France; 10 The Tisch Cancer Institute and Institute for Translational Epidemiology, Mount Sinai School of Medicine, New York, 10029 New York USA; 11 Endocrine Research Center, Research Institute for Endocrine Sciences, Shahid Beheshti University of Medical Sciences, Tehran, 17413 Tehran Iran; 12 Department of Global Health and Population, Harvard School of Public Health, 677 Huntington Avenue, Bldg 1, Boston, 02115 MA USA; 13 Department of Epidemiology, Harvard School of Public Health, 677 Huntington Avenue, Bldg 1, Boston, MA 02115 USA; 14 SAPHIR, the Scientific Association for Public Health in Iran , Boston, 02132 USA

**Keywords:** Incident type 2 diabetes, Refined carbohydrates, White rice, Low- and middle-income countries, Diet

## Abstract

**Background:**

Refined grains and white rice have been associated with elevated risk of type 2 diabetes mellitus (T2DM). In this study, we sought to quantify the effect of white rice intake on incident T2DM in two prospective population-based cohort studies from Iran, where white rice is one of the main staple.

**Methods:**

We used follow-up data from 9,182 participants from Golestan Cohort Study (GCS, 2004–2007, conducted mainly in rural areas) and 2,173 from Tehran Lipid and Glucose Study (TLGS, 2004–2006) who did not have T2DM and other chronic diseases at baseline. Diet was assessed using validated food frequency questionnaires. Multivariable logistic regression models were used to estimate adjusted odds ratios (ORs) for incident T2DM.

**Results:**

We documented 902 new cases of T2DM in GCS and 81 in TLGS. Age-standardized cumulative incidence of T2DM was 9.9% in Golestan and 8.0% in Tehran. Daily white rice intake was significantly higher among residents of Tehran compared to Golestan (median daily intake: 250 vs. 120 grams; *P*-value < 0.001). After adjustment for potential confounders, there was no significant association between daily white rice intake and incident T2DM in GCS. In TLGS, the adjusted OR (95% confidence interval (CI)) was 2.1 (1.1, 3.9) comparing participants with daily white rice intake of >250 grams/day to those with <250.

**Conclusions:**

We observed an increased lieklihood of T2DM associated with high white rice intake among residents of Tehran and no association in Golestan. Our findings, if further supported by other studies, have important public health implications especially for countries where white rice is a major staple and diabetes is increasing rapidly incidence is high. Further research is needed to investigate lack of an association between lower levels of white rice intake and T2DM.

**Electronic supplementary material:**

The online version of this article (doi:10.1186/s12889-016-3999-4) contains supplementary material, which is available to authorized users.

## Background

Prevalence of type 2 diabetes mellitus (T2DM) is increasing globally [1]. Once considered “a disease of affluence”, T2DM is now a worldwide threat with substantially more cases in low- and middle-income countries (LMIC) where resources for prevention, control and management are limited. The Middle East is estimated to have the greatest relative increase in the prevalence of T2DM by 2030 [2]. Prevalence of T2DM in the region was above 10% in 2008 according to the global burden of disease estimates [1]. The prevalence of T2DM among Iranian adults was 8.7% in 2007 based on the third national Surveillance of Risk Factors of Non-Communicable Diseases survey [3] and a 35% increase in diabetes prevalence has been reported between 2005 and 2011 [4]. The prevalence of T2DM for Iran in 2014 was 11.4% (7.2–17.2) according to the latest global burden of disease estimates [5]. Also, the standardized incidence rate of T2DM in adults living in Tehran (above 20 years) was 10.6 per 1000 person-year corresponding to an annual incidence rate of about 1% [6].

Nutritional transition with a more calorie-dense diet and higher consumption of refined carbohydrates (e.g., white rice) may play an important role in the rapid increase in T2DM rates [7–9]. White rice is a staple food in many LMIC facing rapid economic development and nutritional transition. Higher white rice intake has been associated with increased risk of T2DM, particularly in Asian countries [10, 11]. Results of a recent meta-analysis showed a significant 11% increase in risk of T2DM for each additional serving of white rice [12]. In their meta-analysis, Aune et al also reported a 23% increase risk of diabetes with each additional serving of white rice per day [13]. The milling process converts brown rice to white rice removes the majority of fibers, vitamins and minerals of the grain, which have been shown to have anti-diabetic properties [14, 15]. Additionally, white rice has a higher glycemic index (GI) compared to brown rice and other whole grains [16]. High GI diets have been consistently associated with elevated risk of T2DM in several prospective cohort studies [11, 17–19].

Iran is the 13th biggest white rice consumer worldwide with an average annual per capita consumption of an approximately 34 Kg [20]. These high levels of intake in addition to other lifestyle changes such as increased interest in fast and junk food, excessive eating and inadequate physical activity [21, 22] might contribute to the rising rates of T2DM in Iran. However, there has been no analysis of the association between white rice intake and incidence of T2DM in Iran. Therefore, we used data from the Golestan Cohort Study (GCS), conducted in the largely rural province of Golestan, and the Tehran Lipid and Glucose Study (TLGS), conducted in Tehran, to investigate the association between white rice intake and T2DM in populations under different stages of economic development.

## Methods

### Selected cohorts and the study population

Details on the design of both studies have been reported before [23, 24]. GCS was launched in 2004 in Golestan province, Northeast of Iran and recruited 50,045 adults aged between 40 and 87 years from Gonbad city and 326 rural villages (20% urban, 80% rural). Five years after recruitment, a random sample of about 12,000 participants were invited to participate in a re-measurement study. The same questionnaires were filled and all participants were asked to give 30 ml of blood for biomarker measurements including fasting plasma glucose (FPG) and hemoglobin A1c (HbA1c). We used data from the re-measurement sub-sample in this analysis.

Tehran Lipid and Glucose Study (TLGS) enrolled 15,005 participants who were 3 years of age or older residing in the 13th district of Tehran, between 1999 and 2001. Participants are being followed up every 3 years by trained interviewers. Here, we used data from adults 20 years and above who participated in the third (2006–2008) and forth (2009–2011) phases of the study. The follow-up between the third and forth phases of TLGS ranged from 1 to 5 years.

**Fig. 1:**
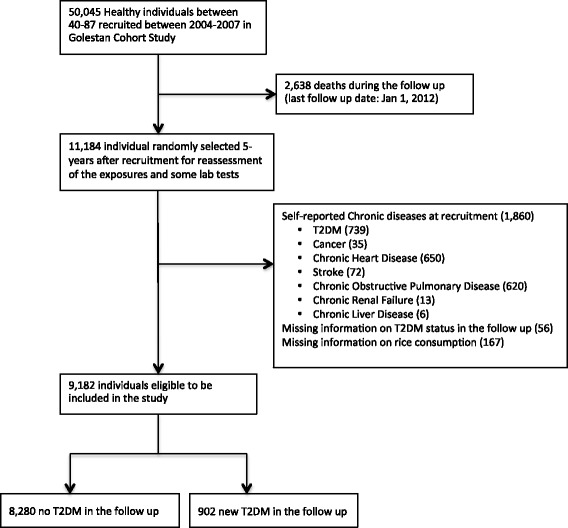
Selection process of eligible participants in Golestan Cohort Study

**Fig. 2:**
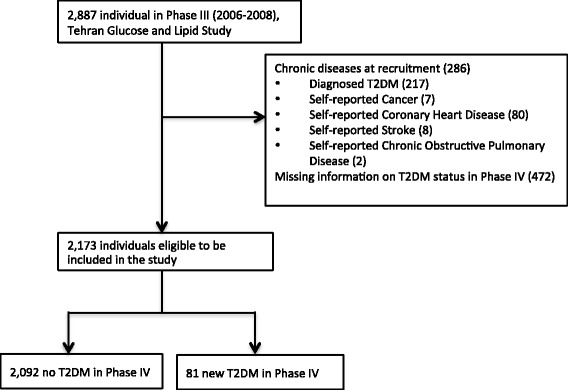
Selection process of eligible participants in Tehran Lipid and Glucose Study

We excluded participants with T2DM (self-reported in GCS and diagnosed with blood measurements or receiving anti-diabetic medications in TLGS), and other non-communicable diseases at baseline (Figs. 1 and 2). We excluded individuals with T2DM at baseline in both studies to ensure individuals with prevalent diabetes are not included in the analyses.

### Exposure assessment

#### GCS

Dietary information was collected using a validated food frequency questionnaire (FFQ) specifically developed for this population [25]. Information on the typical portion size, consumption frequency, and the number of servings consumed each time was collected for each food item at enrollment. Daily intake of each food item was calculated by multiplying the consumption frequency by the typical portion size and the number of servings. We excluded 167 individuals for whom we did not have information on white rice intake. To further investigate the association between white rice intake and T2DM, we divided the participants into quartiles of daily white rice consumption; ≤71.1, 71.2–120, 120.1–210 and >210 grams/day.

#### TLGS

A validated semi-quantitative FFQ was used to collect dietary information in phase III [26]. Trained dietitians with at least 5 years of experience in TLGS asked participants to designate their consumption frequency for each food item consumed during the previous year on a daily, weekly, or monthly basis. Portion sizes of consumed foods that were reported in household measures were then converted to grams [3].

The median white rice intake in this population was 250 grams/day (equivalent of ‘one dish’ of cooked white rice) and 36% of the participants reported this amount as their intake level. Thus, white rice intake in TLGS was categorized into three groups less than 250, 250 and more than 250 grams/day.

### Outcome assessment

GCS participants who had FPG ≥ 126 mg/dl, HbA1c > 6.5% [27] or were receiving treatment for diabetes were categorized as having confirmed incident T2DM. In TLGS phase IV, participants with FPG ≥ 126 mg/dl or 2-h plasma glucose ≥ 200 mg/dl during an oral plasma glucose tolerance test or taking anti-diabetic medications were considered as cases.

### Potential confounders

All participants in both studies underwent interviews by trained physicians and/or technicians and information on demographics and other baseline lifestyle behaviors were collected using structured lifestyle questionnaires. Anthropometric indices were measured after the interviews by trained technicians.

Potential confounders assessed in both studies included age, sex, education (highest level attained), marital status, employment status, smoking, physical activity (physical activity at work estimated from the intensity of the daily work and the amount of time spent at work in GCS and total physical activity calculated from information collected via a validated modifiable activity questionnaire in TLGS) [28]. Our aim was to estimate the effect of substituting white rice with other sources of carbohydrates or fat. Therefore, we adjusted for total calorie intake and daily meat (red meat, poultry and fish) intake. Additional confounders included in the GCS analysis were race (Turkman vs. non-Turkman), urban or rural residence, wealth score (a surrogate of socioeconomic status (SES), calculated from appliance ownership [29]), opium and alcohol consumption and self-reported hypertension status. In TLGS, we also adjusted the models for family history of T2DM.

The GCS was approved by the Institutional Review Boards of the Digestive Disease Research Institute of Tehran University of Medical Sciences, the US National Cancer Institute (NCI), and the World Health Organization International Agency for Research on Cancer (IARC). TLGS was approved by the institutional review board of the Research Institute for Endocrine Sciences, Shahid Beheshti University of Medical Sciences. All participants gave written informed consent before enrollment.

### Statistical analysis

We used the World Standard Population 2000–2005 developed by the WHO [20] to calculate age-standardized rates. Multivariable logistic regression models were used to estimate crude and adjusted odds ratios (ORs) and the corresponding 95% confidence interval (CI)s. Models were initially adjusted for age and sex and further for all other potential confounders mentioned above. Selection of confounders was based on prior knowledge. We examined if the association between white rice intake and T2DM was modified by selected baseline characteristics (age (above or below 50 years), sex and physical activity in both studies and ethnicity and residence in GCS) by including product terms in the models. Since some of the effect of white rice intake on T2DM may be mediated through weight gain, we did not include body mass index (BMI) or waist circumference (WC) in our main analysis.

We used data on demographics and other baseline lifestyle behaviors and dietary information obtained during interview at enrollment in GCS and phase III in TLGS. Laboratory measurements obtained in the re-measurement study in GCS (2011–2013) and fourth phase of TLGS (2009–2011) were used to determine diabetes status.

#### Sensitivity analyses

As adjusting for total calorie intake as a covariate in the model may not be sufficient, in a sensitivity analysis, we used the ‘residual method’ for energy adjustment [30]. In GCS, we first used a Box-Cox power transformation to determine the appropriate exponent (theta = 0.28) that would transform the distribution of white rice intake into a Normal distribution. The residual of the regression of the box-cox transformed white rice intake on total calorie intake was then categorized into quartiles and used as the dependent variable. In TLGS, however, the insufficient number of events in the exposure categories of calorie-adjusted white rice intake limited our ability to perform sensitivity analysis using residual adjustment method.

We further assessed the sensitivity of our results in GCS to selection bias due to differential survival using inverse probability weighting (IPW). This was done mainly since Golestan is an older cohort (average age 51 years old) and we had 2,451 deaths during the follow up. We estimated the probability of death during the 5 years of follow up using a logistic regression model conditional on all the risk factors of both death and T2DM and assigned the reciprocal of the estimated probability to each individual. This approach allows us to create a pseudo-population in which there is no censoring by death (non-stabilized weights) or censoring by death is independent of measured determinants of survival included in the IPW models (stabilized weights) [31]. Finally, we looked at the association between categories of white rice intake and FPG (defined as above 126 mg/dl) in both studies. All tests of hypothesis were conducted at a confidence level of 0.95 under the two-sided alternative.

## Results

A total of 9,182 participants from GCS and 2,173 from TLGS were eligible for this analysis (Figs. 1 and 2). Participants were on average (±SD) 51 (±8) years old at recruitment in GCS and 39 (±13) in TLGS at baseline. We documented 902 diagnosed T2DM in GCS and 81 in TLGS (see Additional file 1: Table S1). Age-standardized cumulative incidence of T2DM was 9.9% in GCS over 5 years and 8.0% in TLGS over three years. Characteristics of study participants by categories of white rice intake in both studies are presented in Tables 1 and 2.

**Table 1:** Baseline Characteristics of Study Participants According to Quartiles of White Rice Intake in Re-measurement Sub-cohort, Golestan Cohort Study (2004–2007)

		≤71.1 (*n* = 2267)	71.2–120 (*n* = 2679)	120.1–210 (*n* = 1990)	>210 (*n* = 2393)	*P*-value
Median rice intake, g/day		51.4	107.1	171.4	257.1	
Diabetes Mellitus diagnosed during re-measurement study, N (%)^a^						
	No	2027 (90.1)	2434 (91.2)	1784 (90.1)	2035 (89.1)	0.114
	Yes	223 (9.9)	235 (8.8)	196 (9.9)	248 (10.2)	
Age Categories, N (%)						
	Below 45	433 (19.1)	637 (23.8)	461 (23.2)	554 (24.2)	<0.001
	45–49	540 (23.8)	664 (24.8)	557 (28.0)	632 (27.6)	
	50–54	467 (20.6)	541 (20.2)	417 (20.9)	463 (20.2)	
	55–59	375 (16.5)	400 (14.9)	274 (13.8)	336 (14.7)	
	60 and above	452 (19.9)	437 (16.3)	281 (14.1)	308 (13.4)	
Female, N (%)		1420 (62.6)	1575 (58.8)	903 (45.4)	856 (37.3)	<0.001
Turkmen, N (%)		1504 (66.3)	2160 (80.6)	1521 (76.4)	1919 (83.7)	<0.001
Rural, N (%)		1892 (83.5)	2208 (82.4)	1475 (74.2)	1718 (74.9)	<0.001
Wealth score, N (%)						
	Low	815 (36.0)	667 (24.9)	390 (19.6)	380 (16.6)	<0.001
	Low-Medium	583 (25.7)	546 (20.4)	362 (18.2)	328 (14.3)	
	Medium-High	515 (22.7)	778 (29.0)	533 (26.8)	601 (26.2)	
	High	354 (15.6)	688 (25.7)	705 (35.4)	984 (42.9)	
Education, N (%)						
	Illiterate	1809 (79.8)	1887 (70.4)	1134 (57.0)	1167 (50.9)	<0.001
	Primary School	303 (13.4)	467 (17.4)	428 (21.5)	542 (23.6)	
	Middle School or Higher	155 (6.8)	325 (12.1)	428 (21.5)	584 (25.5)	
Married, N (%)		1945 (85.8)	2403 (89.7)	1849 (92.9)	2197 (95.8)	<0.001
Currently working, N (%)		1974 (87.1)	2407 (89.8)	1812 (91.1)	2109 (92.0)	<0.001
Tobacco, N (%)						
	Never Smoker	1823 (80.4)	2177 (81.3)	1545 (77.6)	1731 (75.5)	<0.001
	Former Smoker	116 (5.1)	159 (5.9)	148 (7.4)	181 (7.9)	
	Current Smoker	223 (9.9)	246 (9.2)	228 (11.5)	322 (14.0)	
	Ever-hookah, nass or pipe user^b^	105 (4.6)	97 (3.6)	69 (3.5)	59 (2.6)	
Regular Alcohol Consumption, N (%)		48 (2.1)	87 (3.3)	103 (5.2)	131 (5.7)	<0.001
Opium Use, N (%)		360 (15.9)	360 (13.4)	269 (13.5)	320 (14.0)	0.006
Physical Activity, N (%)						
	Mild	1443 (63.6)	1623 (60.6)	1043 (52.4)	1115 (48.6)	<0.001
	Moderate	528 (23.3)	741 (27.6)	695 (34.9)	852 (37.2)	
	Intense	296 (13.1)	315 (11.8)	252 (12.7)	326 (14.2)	
Hypertension, N (%)		947 (41.8)	1059 (39.5)	751 (37.7)	853 (37.2)	0.007
Anthropometric Indices						
Body mass index (BMI) Categories, N (%)						
	Underweight (BMI < 18.5)	137 (6.0)	97 (3.6)	53 (2.7)	43 (1.9)	<0.001
	Normal (20 ≤ BMI < 25)	944 (41.6)	989 (36.9)	643 (32.8)	693 (29.8)	
	Overweight (25 ≤ BMI < 30)	702 (31.0)	927 (34.6)	732 (37.4)	918 (39.5)	
	Obese (BMI ≥ 30)	484 (21.4)	666 (24.9)	532 (27.1)	669 (28.8)	
Waist circumference, Mean (SD)						
Men		90.7 (12.7)	93.0 (12.8)	95.7 (12.8)	98.3 (12.6)	<0.001
Women		93.98 (14.2)	95.83 (14.1)	97.35 (13.0)	98.53 (13.0)	<0.001
Food and Nutrient Intake						
Mean (SD) Carbohydrate Intake, g/day		291.5 (98.6)	308.4 (91.8)	329.2 (90.8)	346.7 (99.1)	<0.001
Mean (SD) Meat Intake, g/day		72.1 (56.0)	76.4 (52.4)	81.9 (51.3)	93.7 (58.5)	<0.001
Mean (SD) Calorie Intake, kCal/day		2024.8 (603.7)	2168.4 (569.1)	2307.7 (565.5)	2456.3 (620.4)	<0.001

^a^The numbers of individuals with and without type 2 diabetes mellitus don’t add up to the total number of participants in some of the categories of white rice consumption due to the missing information of diabetes status on some of the individuals in those categories. ^b^nass: a mixture of tobacco, lime and ash

**Table 2:** Baseline Characteristics of Participants by Categories of White Rice Intake (g/day) in Phase III, Tehran Lipid and Glucose Study (2004–2006)

		<250 (*n* = 876)	250 (*n* = 778)	>250 (*n* = 519)	*P*-value
Median rice intake, g/day		125	250	390	
Diabetes Mellitus diagnosed at phase IV, N (%)^a^					
	No	843 (96.3)	753 (96.8)	496 (95.6)	0.524
	Yes	33 (3.7)	25 (3.2)	23 (4.4)	
Age Categories, N (%)					
	Below 30	198 (22.6)	203 (26.1)	197 (38.0)	<0.001
	30–39	208 (23.7)	211 (27.1)	136 (26.2)	
	40–49	228 (26.0)	175 (22.5)	107 (20.6)	
	50–59	148 (16.9)	111 (14.3)	52 (10.0)	
	60 and above	94 (10.7)	78 (10)	27 (5.2)	
Female, N (%)		564 (64.4)	441 (56.7)	192 (37.0)	<0.001
Education, N (%)					
	High School or lower	264 (30.1)	226 (29.1)	118 (22.7)	0.042
	High School Diploma	393 (44.9)	349 (44.9)	250 (48.2)	
	Above High School Diploma	219 (25.0)	203 (26.1)	151 (29.1)	
Married, N (%)		689 (78.7)	588 (75.6)	364 (70.1)	0.002
Currently working, N (%)		350 (40.0)	356 (45.8)	325 (62.6)	<0.001
Tobacco, N (%)					
	Never Smoker	668 (76.3)	552 (71.0)	323 (62.2)	<0.001
	Former Smoker	49 (5.6)	55 (7.1)	41 (7.9)	
	Current Smoker	159 (18.2)	170 (21.9)	155 (29.9)	
Total Physical Activity, N (%)					
	Light	322 (36.8)	317 (40.8)	227 (43.7)	0.005
	Moderate	217 (24.8)	142 (18.3)	107 (20.6)	
	Vigorous	337 (38.5)	319 (41.0)	185 (35.7)	
Family History of Diabetes, N (%)		166 (19.0)	146 (18.8)	101 (19.5)	0.951
Anthropometric Indices					
Body mass index (BMI) Categories at Baseline, N (%)					
	Underweight (BMI < 18.5)	9 (1.0)	11 (1.4)	3 (0.6)	<0.001
	Normal (20 ≤ BMI < 25)	225 (25.7)	218 (28.0)	171 (33.0)	
	Overweight (25 ≤ BMI < 30)	348 (39.7)	347 (44.6)	222 (42.8)	
	Obese (BMI ≥ 30)	294 (33.6)	202 (26.0)	123 (23.7)	
Waist circumference, Mean (SD)					
Men		95.2 (11.1)	93.8 (10.8)	94.1 (10.8)	0.101
Women		86.3 (13.0)	84.7 (13.3)	83.7 (14.0)	0.015
Food and Nutrient Intake					
Mean (SD) Carbohydrate Intake, g/day		290.6 (126.9)	351.9 (180.8)	448.8 (252.7)	<0.001
Mean (SD) Meat Intake, g/day		57.3 (6.3)	62.3 (52.5)	72.5 (63.8)	<0.001
Mean (SD) Calorie Intake, kCal/day		2072.7 (822.4)	2446.1 (1077.9)	2963.5 (1298.7)	<0.001

^a^The numbers of individuals with and without type 2 diabetes mellitus don’t add up to the total number of participants in some of the categories of white rice consumption due to the missing information of diabetes status on some of the individuals in those categories

Carbohydrate intake accounted for 56.9% and 57.7% of the total daily energy intake GCS and TLGS, respectively. In GCS, white rice was responsible for 8% of total energy intake and 14% of total carbohydrate intake. In TLGS, these figures were 15.7% and 27%, respectively. The median (interquartile range (IQR)) daily white rice intake was 120 grams (77, 210) in GCS and 250 grams (143, 250) in TLGS. Compared to Golestan, residents of Tehran consumed significantly more white rice on a daily basis (*P*-value for Wilcoxon signed-rank test < 0.001). Daily calorie intake mean was also significantly higher in TLGS (2,425 (±1,098) kCal/day in TLGS vs 2231 (±612) kCal/day in GCS; *P*-value < 0.001). Meat, on the other hand, was consumed significantly more in Golestan (80 (±50.3) vs 63 (±53.5) grams/day; *P*-value < 0.001).

In Golestan, non-Turkmens, urban residents, individuals with higher wealth scores, some education, who were currently working, married and were physically more active were more likely to report consuming more than 210 grams of white rice per day. Smoking, alcohol intake, BMI and WC were positively and opium use was negatively associated with white rice intake. In TLGS, marital status and physical activity were negatively and smoking was positively associated with white rice intake. Finally, higher white rice intake was associated with higher daily meat and calorie intake in both studies.

In both crude and fully adjusted models, there was no significant association between white rice and incidence T2DM in GCS (Table 3). In TLGS, the crude OR (95% CI) was 1.18 (0.68, 2.04) and the fully adjusted OR was 2.08 (1.10, 3.91) comparing participants with white rice intake of >250 grams/day to those with <250. The negative confounding pattern is partly due to differences in age and SES where the younger, currently working and more educated participants had higher intake levels (Table 1, Additional file 2: Table S2). The ORs of diabetes obtained from the energy-adjusted white rice intake (residual method) in GCS were almost identical to the above results (see Additional file 3: Table S3). The IPW analysis did not change the ORs of diabetes in GCS materially (see Additional file 3: Table S3).

**Table 3:** Odds ratio (OR) (95% confidence interval (CI)) of type 2 diabetes mellitus according to different categories of white rice intake in Golestan Cohort Study (GCS) and Tehran Lipid and Glucose Study (TLGS), (2004–2007)

	N. Eligible participants	N. Type 2 Diabetes Mellitus	Crude OR (95% CI)	P for trend	Age and sex adjusted OR (95% CI)	P for trend	Fully-adjusted OR (95% CI)	P for trend	Fully-adjusted OR plus Body Mass Index (95% CI)	P for trend
GCS*										
Quartiles of White Rice Intake										
≤ 71.1 g/day	**2,267**	**223**	1	0.14	1	0.02	1	0.42	1	0.54
71.2-120 g/day	**2,679**	**235**	0.88 (0.72, 1.06)		0.90 (0.75, 1.10)		0.89 (0.73, 1.08)		0.85 (0.69, 1.04)	
120.1-210 g/day	**1,990**	**196**	1.00 (0.82, 1.22)		1.07 (0.87, 1.32)		0.94 (0.76, 1.17)		0.84 (0.68, 1.06)	
> 210 g/day	**2,393**	**248**	1.11 (0.92, 1.34)		1.22 (1.01, 1.48)		1.05 (0.85, 1.30)		0.90 (0.73, 1.12)	
TLGS†										
Categories of White Rice Intake										
< 250 g/day	**876**	**33**	1	0.63	1	0.09	1	0.05	1	0.03
250 g/day	**778**	**25**	0.84 (0.50, 1.45)		0.94 (0.55, 1.61)		1.01 (0.58, 1.75)		1.08 (0.61, 1.92)	
> 250 g/day	**519**	**23**	1.18 (0.68, 2.04)		1.78 (1.00, 3.17)		2.08 (1.10, 3.91)		2.28 (1.19, 4.37)	

* Models were adjusted for age categories (below 45, 45–49, 50–54, 55–59, 60 and above), sex (female, male), race/ethnicity (Turkmen, non-Turkmen), wealth score (low, low-medium, medium or high), education (illiterate, primary school, middle school or higher) marital status (single, married), employment status (employed, unemployed), opium (yes, no), alcohol (yes, no), occupational physical activity (mild, moderate, intense), smoking (never, former, current, ever hookah, nass or pipe user), quartiles of daily meat intake (g/d; ≤45, 45.1-69.8, 69.9-102.8, >102.8) and quartiles of daily calorie intake (kcal/d; ≤1840.8, 1840.9-2189.5, 2189.6-2552.4, >2552.4). † Models were adjusted for age categories (below 30, 30–39, 40–9, 50–59, 60 and above), sex (female, male), family history of type 2 diabetes mellitus (yes/no), education (no high school diploma, high school diploma, some university training), marital status (single, married), employment status (employed, unemployed), total physical activity (metabolic equivalent task hours per day categorized as light, moderate, intense), smoking (never, former, current), quartiles of daily meat intake (g/d; ≤32.3, 32.3-50.6, 50.7-76.8, >76.8) and quartiles of daily calorie intake (kcal/d; <1765.1, 1765.1-2237.3, 2237.4-2830.1, >2830.1)

Using FPG as the outcome instead of T2DM, we observed comparable associations in both studies (Additional file 4: Table S4). Similar to T2DM, we observed no association between FPG and white rice intake in GCS. In TLGS, however, the effect of white rice intake on FPG was slightly stronger compared to its effect on T2DM.

Further analyses revealed no modification of the effect in different strata of age, sex, physical activity, race/ethnicity and residence in GCS (Table 4). In TLGS, on the other hand, we observed stronger associations between white rice intake and T2DM in men and individuals with moderate total physical activity (*P* values for trend <0.05). The fully adjusted OR for T2DM for consuming 250 grams/day of white rice was 3.11 (1.23, 7.87) in men (Table 4).

**Table 4:** Odds ratio (OR) (95% confidence interval (CI)) of type 2 diabetes mellitus according to white rice intake by age categories, sex, physical activity, race/ethnicity and residence intake in Golestan Cohort Study (GCS) and Tehran Lipid and Glucose Study (TLGS), (2004–2007)

		GCS*					TLGS†				
		White Rice Intake (grams/day)				P for trend		White Rice Intake (grams/day)			P for trend
	N	≤71.1	71.2-120	120.1-210	>210		N	<250	250	>250	
Age											
Below 50	**4,751**	1	1.03 (0.75, 1.43)	1.22 (0.88, 1.70)	1.10 (0.78, 1.54)	0.11	**1,663**	1	1.54 (0.64, 3.70)	2.40 (0.88, 6.35)	0.11
Above 50	**4,478**	1	0.80 (0.62, 1.03)	0.77 (0.57, 1.02)	1.03 (0.79, 1.36)	0.15	**510**	1	0.75 (0.36, 1.57)	2.02 (0.85, 4.77)	0.20
*P-value for interaction*			0.31	0.04	1.0				0.29	0.89	
Sex											
Female	**4,754**	1	1.00 (0.78, 1.29)	1.03 (0.77, 1.38)	1.11 (0.83, 1.49)	0.53	**1,197**	1	0.88 (0.42, 1.83)	1.52 (0.57, 4.07)	0.71
Male	**4,475**	1	0.70 (0.50, 0.98)	0.85 (0.61, 1.18)	0.95 (0.69, 1.30)	0.56	**976**	1	1.45 (0.58, 3.61)	3.11 (1.23, 7.87)	0.02
*P-value for interaction*			0.12	0.53	0.75				0.36	0.20	
Physical Activity											
Mild	**5,224**	1	1.06 (0.83, 1.35)	0.81 (0.61, 1.09)	1.13 (0.85, 1.50)	0.345	**866**	1	1.04 (0.44, 2.48)	2.22 (0.83, 5.97)	0.17
Moderate	**2,816**	1	0.65 (0.43, 0.98)	1.27 (0.87, 1.87)	1.07 (0.73, 1.57)	0.09	**466**	1	2.50 (0.57, 11.11)	9.86 (2.10, 46.18)	0.01
Intense	**1,189**	1	0.60 (0.31, 1.16)	0.60 (0.30, 1.19)	0.68 (0.35, 1.34)	0.34	**841**	1	0.69 (0.28, 1.69)	0.74 (0.10, 2.56)	0.42
P-value for interaction**			0.79, 0.33	0.04, 0.98	1.0, 0.66				0.29, 0.04	0.54, 0.19	
Race/Ethnicity											
Turkmen	**7,104**	1	0.86 (0.67, 1.09)	0.94 (0.72, 1.22)	1.05 (0.82, 1.36)	0.43					
Non-Turkmen	**2,125**	**1**	0.97 (0.69, 1.38)	0.99 (0.68, 1.45)	1.07 (0.71, 1.61)	0.74					
*P-value for interaction*			0.49	0.75	0.81						
Residence											
Rural	**7,293**	1	0.90 (0.72, 1.13)	0.88 (0.68, 1.14)	1.04 (0.81, 1.34)	0.62					
Urban	**1,936**	1	0.93 (0.60, 1.43)	1.18 (0.77, 1.79)	1.16 (0.76, 1.78)	0.33					
*Pcpovalue for interaction*			0.79	0.59	0.82						

* Models were adjusted for wealth score (low, low-medium, medium or high), education (illiterate, primary school, middle school or higher) marital status (single, married), employment status (employed, unemployed), opium (yes, no), alcohol (yes, no), smoking (never, former, current, ever hookah, nass or pipe user), quartiles of daily meat intake (g/d; ≤45, 45.1-69.8, 69.9-102.8, >102.8) and quartiles of daily calorie intake (kcal/d; ≤1840.8, 1840.9-2189.5, 2189.6-2552.4, >2552.4). Each model was also additionally adjusted for other covariates not stratified for. † Models were adjusted for family history of type 2 diabetes mellitus (yes/no), education (no high school diploma, high school diploma, some university training), marital status (single, married), employment status (employed, unemployed), smoking (never, former, current), family history of diabetes mellitus (yes, no), quartiles of daily meat intake (g/d; ≤32.3, 32.3-50.6, 50.7-76.8, >76.8) and quartiles of daily calorie intake (kcal/d; <1765.1, 1765.1-2237.3, 2237.4-2830.1, >2830.1). Each model was also additionally adjusted for other covariates not stratified for. **Two P-values for interactions are presented; the first represent P-values for the interactions between moderate physical activity and categories of white rice consumption and the second represent p-values for the interaction between intense physical activity and categories of white rice intake

## Discussion

In these two prospective population-based cohort studies of rural and urban areas in Iran, we observed an association between white rice intake and T2DM only in urban areas. While we detected a significant doubling of incidence of diabetes in those with white rice intake more than 250 grams/day in TLGS, in Tehran, we did not observe a significant association in the GCS, in Golestan. The observed association in TLGS was more pronounced in men.

Several prospective studies have reported a positive association between white rice intake and diabetes [10, 11, 32]. Hu et al. conducted a meta-analysis on seven prospective cohort studies conducted in Asian and Western populations and observed an overall 27% increased risk of diabetes comparing high versus low white rice consumption levels. This effect was more pronounced in Asian populations that consume white rice at larger quantities (55% increase risk) [12]. We observed a doubling of diabetes risk associated with >250 grams of white rice intake in TLGS. This finding is in line with but stronger than the results of the prospective Asian studies. The Shanghai Women’s Health Study reported a 78% higher risk of diabetes with >300 grams/day of raw white rice intake (compared to less than 200 grams of raw white rice intake) [11] and the Japanese Public Health Center-based Prospective Study (Women) showed 65% increase in diabetes risk with consuming >437 grams of cooked white rice each day compared to comsuming <287 grams of daily cooked white rice [10]. Similarly, regular white rice intake was associated with increased risk and replacement of white rice with whole grain was associated with a reduced risk of diabetes in US men and women [32]. The differences in the effect of white rice on diabetes across various populations can be due to different carbohydrate quantity and quality. The effect of white rice on diabetes could be more pronounced in developing countries undergoing nutritional transition. Fat accumulation through increased intake of calorie-dense diet, and lack of physical activity could make people living in these countries more susceptible to the adverse effects of higher white rice intake.

The exact mechanism through which white rice intake may increase the risk of diabetes is not clear, but postprandial blood glucose surge (measured by the GI) and further induction of insulin resistance has been implicated as a possible mechanism [33]. Both the GI and glycemic load have been associated with increased risk of metabolic syndrome and diabetes in several prospective cohort studies [11, 17–19]. The GI of white rice depends on the proportion of the amylose content of the grain in addition to the cooking time and the degree of processing [16]. But in general, the GI value of white rice has been reported to be higher than that of brown rice and other whole grains [16]. In addition, the milling process removes the outer bran layer of the grain which contains important nutrients such as magnesium and fibers which have been associated with decreased risk of diabetes [14, 15].

The contribution of white rice to the total energy intake was generally low in both studies analyzed here (8% in GCS and 15.7% in TLGS), while carbohydrate accounts for approximately 60% of the total daily energy intake. This indicates that other foods containing high carbohydrates (such as bread) are being consumed at high quantities in these populations. Bread is considered a main staple in Golestan and the median daily bread intake in Golestan was 352 grams, which accounted for approximately 52% of total carbohydrate and 30% of total calorie intake.

The discrepancy between the results of the GCS and TLGS may be due to (1) lower levels of exposure in the GCS; (2) effect measure modification; or (3) differences in measurement or residual confounding between the two studies. On average, GCS participants consumed 97 grams/day less white rice and white rice only contributed to 14% of the total daily carbohydrate intake (compared to 27% in TLGS). It is possible that higher consumption levels are required for white rice to affect diabetes risk [12]. The GCS was conducted in Golestan province, a predominantly rural province which is less developed economically than major cities such as Tehran. This may lead to different levels of lifestyle and environmental factors that may act as effect modifiers. Specifically, it is also possible that GCS participants are consuming more of other forms of carbohydrates, namely bread, that are richer in insoluble fibers and magnesium, and the beneficial anti-diabetic effects of these nutrients could potentially cancel out the adverse effect of white rice. Additionally, GCS participants are mostly farmers and are more likely to have an active lifestyle compared to residents of Tehran, who are mostly white-collar workers. Physical activity was not measured with the same tool in both studies so we could not directly compare physical activity levels between the two cohorts. A specific source of bias in the GCS is that blood glucose was not measured at baseline so some of the diabetes cases in our analysis may indeed be prevalent cases and this will bias the estimates towards the null. We also expect larger residual or unmeasured confounding by SES in TLGS compared to the GCS as the only SES variable available in TLGS was education.

This study has some limitations. First, information on the date of diagnosis of diabetes was not available in either study, so we could not conduct a time-to-event analysis. Second, measurement errors in dietary intake elicited using FFQs cannot be ruled out, even though both FFQs have been previously shown to provide reliable and valid estimates of nutrients and energy intake [25, 26]. It is possible the FFQs used in the two studies have different accuracy white rice intake measurement. However, validity and reliability of both FFQs have been shown to be comparable [25, 26], so the difference in measurement errors was less likely to explain the observed difference in the association between T2DM and white rice intake. Additionally, dietary information was gathered before diabetes diagnosis in both studies. Thus, any measurement error in white rice intake would be non-differential. Although we have previously shown a notable agreement between self-reported diabetes and diabetes as defined by measurements of blood glucose [34], the self-reported nature of diabetes assessment at baseline in Golestan could have led to overestimation of diabetes incidence in this population. Third, the relatively small sample size in TLGS affected the reliability of some of the estimates from the stratified analyses (i.e., physical activity). Finally, the possibility of residual confounding by unmeasured confounders or measurement error in measured confounders cannot be ruled out.

Some of the strengths of this study include its large sample size, accurate outcome assessment (FPG in both studies, HbA1c in GCS and 2-h plasma glucose in TLGS) and available data on many potential confounders as well as the opportunity to assess the association between white rice intake and diabetes in urban and rural populations in the Middle East.

## Conclusion

In summary, we observed an increased likelihood of T2DM associated with high white rice intake among residents of Tehran and no association in Golestan. Our findings, if further supported by other studies, have important public health implications for other countries in the Middle East and North Africa as well as countries in South, East and South East Asia where white rice is a major staple and diabetes is increasing rapidly [1]. Introducing whole grains and replacing white rice with whole grain carbohydrates can be one of the strategies for prevention of T2DM. Our study was a step towards investigating the role of refined carbohydrates on incidence of T2DM in an area with increasing rates of the disease. Future research should focus on assessing the impact of other commonly used refined carbohydrate including bread, especially in rural areas and in areas with lower levels of white rice intake.

## Additional files

Additional file 1:
**Table S1.** Distribution of individuals in Golestan Cohort Study (GCS) and Tehran Lipid and Glucose Study (TLGS) meeting fasting plasma glucose (FPG), hemoglobin A1c (HbA1c), oral glucose tolerance test (OGTT) and diabetes medications criterion for Type 2 Diabetes Mellitus Definition. (DOCX 46 kb)
Additional file 2:
**Table S2.** OR (95% CI) of diabetes mellitus according to white rice intake by age categories, sex, physical activity, race/ethnicity and residence intake in Tehran Lipid and Glucose Study (TLGS), (2004–2006). (DOCX 72 kb)
Additional file 3: Table S3. Un-weighted and weighted OR (95% CI) of diabetes mellitus according to white rice intake in the Golestan Cohort Study (GCS), (2004–2007)* (DOCX 19 kb)
Additional file 4:
**Table S4.** OR (95% CI) of fasting plasma glucose above 126 mg/dl according to different categories of white rice intake in Golestan Cohort Study (GCS) and Tehran Lipid and Glucose Study (TLGS), (2004–2007). (DOCX 88 kb)

## Abbreviations

CI: Confidence interval
FFQ: Food frequency questionnaire
FPG: Fasting plasma glucose
GCS: Glolestan Cohort Study
GI: Glycemic index
IPW: Inverse probability weighting
OR: Odds ratio
SES: Socioeconomic status
T2DM: Type 2 diabetes mellitus
TLGS: Tehran Lipid and Glucose Study

## References

[1] Danaei G, Finucane MM, Lu Y, Singh GM, Cowan MJ, Paciorek CJ, Lin JK, Farzadfar F, Khang YH, Stevens GA (2011). National, regional, and global trends in fasting plasma glucose and diabetes prevalence since, 1980: systematic analysis of health examination surveys and epidemiological studies with 370 country-years and 2.7 million participants. Lancet.

[2] Hossain P, Kawar B, El Nahas M (2007). Obesity and diabetes in the developing world--a growing challenge. N Engl J Med.

[3] Esteghamati A, Meysamie A, Khalilzadeh O, Rashidi A, Haghazali M, Asgari F, Kamgar M, Gouya MM, Abbasi M (2009). Third national Surveillance of Risk Factors of Non-Communicable Diseases (SuRFNCD-2007) in Iran: methods and results on prevalence of diabetes, hypertension, obesity, central obesity, and dyslipidemia. BMC Public Health.

[4] Esteghamati A, Etemad K, Koohpayehzadeh J, Abbasi M, Meysamie A, Noshad S, Asgari F, Mousavizadeh M, Rafei A, Khajeh E (2014). Trends in the prevalence of diabetes and impaired fasting glucose in association with obesity in Iran: 2005–2011. Diabetes Res Clin Pract.

[5] Collaboration NCDRF (2016). Worldwide trends in diabetes since 1980: a pooled analysis of 751 population-based studies with 4.4 million participants. Lancet.

[6] Harati H, Hadaegh F, Saadat N, Azizi F (2009). Population-based incidence of Type 2 diabetes and its associated risk factors: results from a six-year cohort study in Iran. BMC Public Health.

[7] Finucane MM, Stevens GA, Cowan MJ, Danaei G, Lin JK, Paciorek CJ, Singh GM, Gutierrez HR, Lu Y, Bahalim AN (2011). National, regional, and global trends in body-mass index since 1980: systematic analysis of health examination surveys and epidemiological studies with 960 country-years and 9.1 million participants. Lancet.

[8] Ghassemi H, Harrison G, Mohammad K (2002). An accelerated nutrition transition in Iran. Public Health Nutr.

[9] Kilpi F, Webber L, Musaigner A, Aitsi-Selmi A, Marsh T, Rtveladze K, McPherson K, Brown M (2014). Alarming predictions for obesity and non-communicable diseases in the Middle East. Public Health Nutr.

[10] Nanri A, Mizoue T, Noda M, Takahashi Y, Kato M, Inoue M, Tsugane S (2010). Rice intake and type 2 diabetes in Japanese men and women: the Japan Public Health Center-based Prospective Study. Am J Clin Nutr.

[11] Villegas R, Liu S, Gao YT, Yang G, Li H, Zheng W, Shu XO (2007). Prospective study of dietary carbohydrates, glycemic index, glycemic load, and incidence of type 2 diabetes mellitus in middle-aged Chinese women. Arch Intern Med.

[12] Hu EA, Pan A, Malik V, Sun Q (2012). White rice consumption and risk of type 2 diabetes: meta-analysis and systematic review. BMJ.

[13] Aune D, Norat T, Romundstad P, Vatten LJ (2013). Whole grain and refined grain consumption and the risk of type 2 diabetes: a systematic review and dose-response meta-analysis of cohort studies. Eur J Epidemiol.

[14] Slavin JL, Martini MC, Jacobs DR, Marquart L (1999). Plausible mechanisms for the protectiveness of whole grains. Am J Clin Nutr.

[15] Schulze MB, Schulz M, Heidemann C, Schienkiewitz A, Hoffmann K, Boeing H (2007). Fiber and magnesium intake and incidence of type 2 diabetes: a prospective study and meta-analysis. Arch Intern Med.

[16] Foster-Powell K, Holt SH, Brand-Miller JC (2002). International table of glycemic index and glycemic load values: 2002. Am J Clin Nutr.

[17] Oba S, Nanri A, Kurotani K, Goto A, Kato M, Mizoue T, Noda M, Inoue M, Tsugane S (2013). Dietary glycemic index, glycemic load and incidence of type 2 diabetes in Japanese men and women: the Japan Public Health Center-based Prospective Study. Nutr J.

[18] Salmeron J, Ascherio A, Rimm EB, Colditz GA, Spiegelman D, Jenkins DJ, Stampfer MJ, Wing AL, Willett WC (1997). Dietary fiber, glycemic load, and risk of NIDDM in men. Diabetes Care.

[19] Salmeron J, Manson JE, Stampfer MJ, Colditz GA, Wing AL, Willett WC (1997). Dietary fiber, glycemic load, and risk of non-insulin-dependent diabetes mellitus in women. JAMA.

[20] IRRI in Iran. http://ricetoday.irri.org/irri-in-iran/. Accessed Feb 2014.

[21] Habibzadeh F (2012). Diabetes in the Middle East. Lancet.

[22] Malekzadeh RM, Merat M, Pourshams S, Etemadi A (2005). Obesity Panedemic: an Iranian Perspective. Arch Iranian Med.

[23] Azizi F, Ghanbarian A, Momenan AA, Hadaegh F, Mirmiran P, Hedayati M, Mehrabi Y, Zahedi-Asl S (2009). Prevention of non-communicable disease in a population in nutrition transition: Tehran Lipid and Glucose Study phase II. Trials.

[24] Pourshams A, Khademi H, Malekshah AF, Islami F, Nouraei M, Sadjadi AR, Jafari E, Rakhshani N, Salahi R, Semnani S (2010). Cohort Profile: The Golestan Cohort Study-a prospective study of oesophageal cancer in northern Iran. Int J Epidemiol.

[25] Malekshah AF, Kimiagar M, Saadatian-Elahi M, Pourshams A, Nouraie M, Goglani G, Hoshiarrad A, Sadatsafavi M, Golestan B, Yoonesi A (2006). Validity and reliability of a new food frequency questionnaire compared to 24 h recalls and biochemical measurements: pilot phase of Golestan cohort study of esophageal cancer. Eur J Clin Nutr.

[26] Mirmiran P, Esfahani FH, Mehrabi Y, Hedayati M, Azizi F (2010). Reliability and relative validity of an FFQ for nutrients in the Tehran lipid and glucose study. Public Health Nutr.

[27] Diagnosis and classification of diabetes mellitus. Diabetes Care 2014, 37 Suppl 1:S81-90. 10.2337/dc14-S081 24357215

[28] Momenan AA, Delshad M, Sarbazi N, Rezaei Ghaleh N, Ghanbarian A, Azizi F (2012). Reliability and validity of the Modifiable Activity Questionnaire (MAQ) in an Iranian urban adult population. Arch Iran Med.

[29] Islami F, Kamangar F, Nasrollahzadeh D, Aghcheli K, Sotoudeh M, Abedi-Ardekani B, Merat S, Nasseri-Moghaddam S, Semnani S, Sepehr A (2009). Socio-economic status and oesophageal cancer: results from a population-based case-control study in a high-risk area. Int J Epidemiol.

[30] Willett W (2012). Nutritional Epidemiology (Monographs in Epidemiology and Biostatistics).

[31] Cole SR, Hernan MA (2008). Constructing inverse probability weights for marginal structural models. Am J Epidemiol.

[32] Sun Q, Spiegelman D, van Dam RM, Holmes MD, Malik VS, Willett WC, Hu FB (2010). White rice, brown rice, and risk of type 2 diabetes in US men and women. Arch Intern Med.

[33] Yu D, Shu XO, Li H, Xiang YB, Yang G, Gao YT, Zheng W, Zhang X (2013). Dietary carbohydrates, refined grains, glycemic load, and risk of coronary heart disease in Chinese adults. Am J Epidemiol.

[34] Golozar A, Khademi H, Kamangar F, Poutschi H, Islami F, Abnet CC, Freedman ND, Taylor PR, Pharoah P, Boffetta P (2011). Diabetes mellitus and its correlates in an Iranian adult population. PLoS One.

