# Supplementary material for: White rice intake and incidence of type-2 diabetes: analysis of two prospective cohort studies from Iran
Source: BMC Public Health. 2017 Jan 31;17:133. doi: 10.1186/s12889-016-3999-4 (PMC5282785; doi:10.1186/s12889-016-3999-4)
Supplement: Additional file 1: — Table S1. Distribution of individuals in Golestan Cohort Study (GCS) and Tehran Lipid and Glucose Study (TLGS) meeting fasting plasma glucose (FPG), hemoglobin A1c (HbA1c), oral glucose tolerance test (OGTT) and diabetes medications criterion for Type 2 Diabetes Mellitus Definition. (DOCX 46 kb) [file 12889_2016_3999_MOESM1_ESM.docx]

| **Table S1. Distribution of individuals in Golestan Cohort Study (GCS) and Tehran Lipid and Glucose Study (TLGS) meeting fasting plasma glucose (FPG), hemoglobin A1c (HbA1c), oral glucose tolerance test (OGTT) and diabetes medications criterion for Type 2 Diabetes Mellitus Definition.** | | |
| --- | --- | --- |
|  | **Golestan Cohort Study (GCS) (N=9323)** | **Tehran Lipid and Glucose Study (TLGS) (N=2173)** |
| **Fasting Plasma Glucose≥126 mg/dL, N (%)** | 698 (7.49) | 45 (2.09) |
| **HbA1c≥6.5, N (%)** | 473 (5.07) | --- |
| **Diabetes medication, N (%)** | 317 (3.40) | 14 (0.54) |
| **2-hour plasma glucose≥200 mg/dl, N (%)** | --- | 48 (2.30) |
| **Type 2 Diabetes Mellitus, N (%)** | 902 (9.82) | 81 (3.11) |
