# Supplementary material for: White rice intake and incidence of type-2 diabetes: analysis of two prospective cohort studies from Iran
Source: BMC Public Health. 2017 Jan 31;17:133. doi: 10.1186/s12889-016-3999-4 (PMC5282785; doi:10.1186/s12889-016-3999-4)
Supplement: Additional file 2: — Table S2. OR (95% CI) of diabetes mellitus according to white rice intake by age categories, sex, physical activity, race/ethnicity and residence intake in Tehran Lipid and Glucose Study (TLGS), (2004–2006). (DOCX 72 kb) [file 12889_2016_3999_MOESM2_ESM.docx]

| **Table S2. Odds ratio (OR) (95% confidence interval (CI)) of type 2 diabetes mellitus according to white rice intake by age categories, sex, physical activity, race/ethnicity and residence intake in Tehran Lipid and Glucose Study (TLGS), (2004-2006)** | | | | | | | |
| --- | --- | --- | --- | --- | --- | --- | --- |
|  |  | | **TLGS†** | | | | |
|  |  | |  | **White Rice Intake (grams/day)** | | | **P for trend** |
|  |  | | **N** | **<250 g/day** | **250 g/day** | **>250 g/day** |  |
| **Age** | | |  |  |  |  |  |
|  | **Below 50** | | **1,663** | 1 | 1.54 (0.64, 3.70) | 2.40 (0.88, 6.35) | 0.11 |
|  | **Above 50** | | **510** | 1 | 0.75 (0.36, 1.57) | 2.02 (0.85, 4.77) | 0.20 |
| **Education** | | |  |  |  |  |  |
|  | **High School or lower** | | **590** | 1 | 0.71 (0.31, 1.64) | 2.67 (1.03, 6.95) | 0.19 |
|  | **High School Diploma** | | **981** | 1 | 1.07 (0.42, 2.77) | 1.42 (0.45, 4.46) | 0.71 |
|  | **Above High School Diploma** | | **566** | 1 | 4.05 (0.74, 22.12) | 5.07 (0.80, 32.17) | 0.05 |
| **Currently working** | |  |  | |  |  |  |
|  | **Mild** | | **1,120** | 1 | 0.76 (0.37, 1.6) | 2.20 (0.95, 5.10) | 0.24 |
|  | **Moderate** | | **1,017** | 1 | 1.70 (0.65, 4.44) | 2.37 (0.85, 6.60) | 0.11 |
| * Models were adjusted for family history of type 2 diabetes mellitus (yes/no), education (no high school diploma, high school diploma, some university training), marital status (single, married), employment status (employed, unemployed), smoking (never, former, current), family history of diabetes mellitus (yes, no), quartiles of daily meat intake (g/d; ≤32.3, 32.3-50.6, 50.7-76.8, >76.8) and quartiles of daily calorie intake (kcal/d; <1765.1, 1765.1-2237.3, 2237.4-2830.1, >2830.1). Each model was also additionally adjusted for other covariates not stratified for. | | | | | | | |
