# Supplementary material for: White rice intake and incidence of type-2 diabetes: analysis of two prospective cohort studies from Iran
Source: BMC Public Health. 2017 Jan 31;17:133. doi: 10.1186/s12889-016-3999-4 (PMC5282785; doi:10.1186/s12889-016-3999-4)
Supplement: Additional file 3: Table S3. — Un-weighted and weighted OR (95% CI) of diabetes mellitus according to white rice intake in the Golestan Cohort Study (GCS), (2004–2007)* (DOCX 19 kb) [file 12889_2016_3999_MOESM3_ESM.docx]

| **Table S3. Un-weighted and weighted odds ratio (OR) (95% confidence interval (CI)) of type 2 diabetes mellitus according to white rice intake in the Golestan Cohort Study (GCS), (2004-2007)*** | | | | |
| --- | --- | --- | --- | --- |
|  |  | **Unweighted Regression** | **Weighted Regressions†** | |
|  |  |  | **Not Stabilized weights**‡ | **Stabilized Weights**‡ |
|  |  | Adjusted OR (95% CI) | OR (95% CI)§ | OR (95% CI)§ |
| **Quartiles of White Rice Consumption** | |  |  |  |
|  | **≤71.1 g/day** | 1 | 1 | 1 |
|  | **71.2-120 g/day** | 0.89 (0.72, 1.09) | 0.87 (0.71, 1.07) | 0.89 (0.72, 1.09) |
|  | **120.1-210 g/day** | 0.94 (0.76, 1.17) | 0.94 (0.76, 1.16) | 0.94 (0.76, 1.17) |
|  | **>210 g/day** | 1.05 (0.85, 1.30) | 1.05 (0.85, 1.29) | 1.05 (0.85, 1.30) |
|  | ***P-value for trend*** | 0.42 | 0.40 | 0.42 |
| **Quartiles of the Residuals (Residual Model)** | | |  |  |
|  | **Q1** | 1 | 1 | 1 |
|  | **Q2** | 0.97 (0.80, 1.19) | 0.97 (0.80, 1.19) | 0.97 (0.80, 1.19) |
|  | **Q3** | 0.95 (0.77, 1.16) | 0.95 (0.77, 1.17) | 0.95 (0.77, 1.16) |
|  | **Q4** | 1.06 (0.87, 1.30) | 1.07 (0.87, 1.31) | 1.06 (0.87, 1.30) |
|  | ***P-value for trend*** | 0.57 | 0.53 | 0.57 |
| * Models were adjusted for age categories (below 45, 45-49, 50-54, 55-59, 60 and above), sex (female, male), race/ethnicity (Turkmen, non-Turkmen), wealth score (low, low-medium, medium or high), education (illiterate, primary school, middle school or higher) marital status (single, married), employment status (employed, unemployed), opium (yes, no), alcohol (yes, no), occupational physical activity (mild, moderate, intense), smoking (never, former, current, ever hookah, nass or pipe user), quartiles of daily meat intake (g/d; ≤45, 45.1-69.8, 69.9-102.8, >102.8) and quartiles of daily calorie intake (kcal/d; ≤1840.8, 1840.9-2189.5, 2189.6-2552.4, >2552.4). †Inverse probability weighting for death during the follow up ‡ mean (SD) were 4.0 (0.5) and 1.0 (0.07) for not stabilized and stabilized weights for quartiles of white rice intake and were 4.0 (0.1) and 1.0 (0.03) for not stabilized and stabilized weights for quartiles of the residuals, respectively. § Robust CI | | | | |
|  | | | | |
