# Supplementary material for: White rice intake and incidence of type-2 diabetes: analysis of two prospective cohort studies from Iran
Source: BMC Public Health. 2017 Jan 31;17:133. doi: 10.1186/s12889-016-3999-4 (PMC5282785; doi:10.1186/s12889-016-3999-4)
Supplement: Additional file 4: — Table S4. OR (95% CI) of fasting plasma glucose above 126 mg/dl according to different categories of white rice intake in Golestan Cohort Study (GCS) and Tehran Lipid and Glucose Study (TLGS), (2004–2007). (DOCX 88 kb) [file 12889_2016_3999_MOESM4_ESM.docx]

| **Table S4. Odds ratio (OR) (95% confidence interval (CI)) of fasting plasma glucose above 126 mg/dl according to different categories of white rice intake in Golestan Cohort Study (GCS) and Tehran Lipid and Glucose Study (TLGS), (2004-2007)** | | | | | | | | | | | |
| --- | --- | --- | --- | --- | --- | --- | --- | --- | --- | --- | --- |
|  | |  | | **N. Eligible participants** | **N. Type 2 Diabetes Mellitus** | **Crude OR (95% CI)** | **P for trend** | **Age and sex adjusted OR (95% CI)** | **P for trend** | **Fully-adjusted OR (95% CI)** | **P for trend** |
| **GCS*** | |  | |  |  |  |  |  |  |  |  |
| **Quartiles of White Rice Intake** | | | |  |  |  |  |  |  |  |  |
|  | | **≤71.1 g/day** | | **2,267** | **223** | 1 | 0.14 | 1 | 0.06 | 1 | 0.86 |
|  | | **71.2-120 g/day** | | **2,679** | **235** | 0.91 (0.73, 1.13) |  | 0.93 (0.75, 1.15) |  | 0.84 (0.71, 1.11) |  |
|  | | **120.1-210 g/day** | | **1,990** | **196** | 0.94 (0.75, 1.19) |  | 0.98 (0.78, 1.24) |  | 0.84 (0.66, 1.08) |  |
|  | | **>210 g/day** | | **2,393** | **248** | 1.15 (0.93, 1.43) |  | 1.21 (0.97, 1.51) |  | 1.01 (0.79, 1.28) |  |
| **TLGS†** | |  | |  |  |  |  |  |  |  |  |
| **Categories of White Rice Intake** | | | |  |  |  |  |  |  |  |  |
|  | | **<250 g/day** | | **876** | **33** | 1 | 0.19 | 1 | 0.05 | 1 | 0.04 |
|  | | **250 g/day** | | **778** | **25** | 1.08 (0.53, 2.23) |  | 1.15 (0.56, 2.40) |  | 1.22 (0.58, 2.58) |  |
|  | | **>250 g/day** | | **519** | **23** | 1.64 (0.79, 3.38) |  | 2.24 (1.05, 4.79) |  | 2.57 (1.10, 5.73) |  |
| * Models were adjusted for age categories (below 45, 45-49, 50-54, 55-59, 60 and above), sex (female, male), race/ethnicity (Turkmen, non-Turkmen), wealth score (low, low-medium, medium or high), education (illiterate, primary school, middle school or higher) marital status (single, married), employment status (employed, unemployed), opium (yes, no), alcohol (yes, no), occupational physical activity (mild, moderate, intense), smoking (never, former, current, ever hookah, nass or pipe user), quartiles of daily meat intake (g/d; ≤45, 45.1-69.8, 69.9-102.8, >102.8) and quartiles of daily calorie intake (kcal/d; ≤1840.8, 1840.9-2189.5, 2189.6-2552.4, >2552.4). † Models were adjusted for age categories (below 30, 30-39, 40-9, 50-59, 60 and above), sex (female, male), family history of type 2 diabetes mellitus (yes/no), education (no high school diploma, high school diploma, some university training), marital status (single, married), employment status (employed, unemployed), total physical activity (metabolic equivalent task hours per day categorized as light, moderate, intense), smoking (never, former, current), quartiles of daily meat intake (g/d; ≤32.3, 32.3-50.6, 50.7-76.8, >76.8) and quartiles of daily calorie intake (kcal/d; <1765.1, 1765.1-2237.3, 2237.4-2830.1, >2830.1). | | | | | | | | | | | |
|  |  | |  | | | | | | | | |
